# 

**Published:** 1899-05-13

**Authors:** 


					CADBURYS m* j|> k, OADBURY'S
coooa IL M m JPL, cocoa
ABSOLUTELY PURE. NO DRUGS OR ALKALIES USED.
No. 65;
\
May 13th, 1899.
[bubsonpti?n lenna,] pRICI; TWOPENCE.

				

